# Annotations

**Published:** 1900-04-21

**Authors:** 


### Annotations.

### The Welfare of the Feeble-Minded.

The National Association for Promoting the Welfare of the Feeble-minded has published its third annual report, an interesting document, which shows what has been done for a peculiarly helpless class of the community, and how much good has been attained by it. There are now 17 homes for the feeble-minded in England, 15 of which belong to the Union of Homes of this association. Children are taken into these homes from the age of seven, and are kept and educated until they can safely be placed in situations carefully selected with a view to their limitations. Such as are permanently unfit to battle with the world are employed in some way or another about the homes. But many who under ordinary circumstances seemed to be wholly unintelligent and evil-natured have, after the training they received in the homes, been fit to go into domestic service. Other girls go out to daily work, but sleep at the home, thus continuing to receive the care and discipline their mental condition needs. A few children have been rejected as being too weak mentally and physically to profit by the instruction received; but in many cases where parents and guardians had no hope of the child being capable of learning, there has been a considerable development of intelligence. While manual training is a feature of the instruction in all the homes, the more intellectual parts of education are not neglected, though the method, if not the matter of instruction, is rather that of the infant room than of the senior classes in an ordinary school. There is no doubt that this association does a useful work in training to usefulness many children who would, if neglected spend lives full of misery and disappointment, and who, with their weakness of intellect, are peculiarly liable to be led into vice and crime.

### The Midwives Bill.

After various vicissitudes, and after very nearly meeting with unexpected success, the Midwives Bill seems now to be safely shelved until after Whitsuntide, and if any organised attempt is to be made to improve it now is the time. We are afraid, however, that, in view of the extreme lack of unanimity which seems to exist among those medical men who have taken the trouble to consider the matter seriously, and of the indifference to the whole subject displayed by the majority, no individual attempt to influence members of Parliament will do much good. Indeed, unless we are all prepared to pull in the same direction it may be doubted whether the irresponsible advice of many counsellors may not tend to detract from the weight which ought to attach to the recommendation which we may expect will in due course be given by those official bodies which may properly be regarded as speaking in the name of the profession. There are three public bodies in England from which Parliament may well expect to receive official counsel in the dilemma—the General Medical Council, the Royal College of Physicians, and the Royal College of Surgeons—and in addition to these there is one private body, the British Medical Association, which includes in its membership so large a proportion of the medical profession that it may fairly be asked to join in advising what should be done; and we cannot but think that, disjointed as the profession appears to be, if these four bodies were to show themselves fairly unanimous in recommending clauses having for their object the honest improvement and not the wrecking of the Bill, a measure might be passed which would afford all the protection to parturient women which is sought to be provided, and at the same time would avoid the evils which might arise from the passing of an ill-considered measure. Unfortunately many members of our profession have given just cause for the belief that on this question they are irreconcilable, and there can be no doubt that the position of antagonism so taken up adds greatly to the difficulty of obtaining a proper hearing for moderate and useful amendments.

### Fees for Certificates.

On the plea that the section of the Lunacy Act of 1890, which refers to the certification of paupers in workhouses, says that the Guardians "shall pay such reasonable remuneration as they think fit to the medical officer who, not being an officer of the workhouse, examines persons for the purpose of certifying," under the particular section in question, the Guardians of the Chertsey Union appear to have considered that after Dr. Hope had examined their pauper lunatics, and given the certificates which were required by the Act, they were quite at liberty to give him in return whatever fee they chose. As it happened they thought fit to regard one-half the usual guinea fee as "reasonable remuneration." Not unnaturally Dr. Hope did not agree with them, and on their refusing to pay more appealed to the County Court. The result was that the doctor got his fee, and the matter is of especial interest from the fact that this was a case in which a number of paupers were brought forward to be certified *en bloc*, and that if the retail dealer's plan of giving "a reduction on taking a quantity" had been admitted as applicable to the case of certifying seven paupers in a row, not only would medical men be deprived of their proper remuneration, but the public would lose much of the protection and security which it has been the special object of the various Lunacy Acts to provide. The very object of these laws is to prevent persons being dealt with as lunatics unless they are independently examined and certified, and if it were to be admitted that paupers might be certified *en bloc* at half-price, the temptation would be strong for guardians to save up their lunatics; in other words, to detain them illegally in order that they might get a lot through together. Plenty of evidence, however, was produced, both from medical men and from clerks to guardians and others, to show that a guinea was the ordinary fee for an examination and certificate; and as there was no proof of any other arrangement having been made between the guardians and Dr. Hope he got his case. His Honour, in giving judgment, pointed out the responsibility which attached to the giving of these certificates, and the great interest which the public had in all such examinations and certification being properly done. "It did not matter," he said, "whether the examination took five minutes or 100 minutes. It was not a matter of time, but of professional judgment and skill and experience." This is evidently the proper way in which to regard the question of professional remuneration, and Dr. Hope is to be congratulated on having been the occasion of such a definite pronouncement. Still more, however, are the public, and especially the poor, to be congratulated on the fact that guardians have not been allowed to get off "on the cheap" because they have brought up their lunatics to be certified, as their counsel described it, *en bloc*.

